# Bacteriophages Could Be a Potential Game Changer in the Trajectory of Coronavirus Disease (COVID-19)

**DOI:** 10.1089/phage.2020.0014

**Published:** 2020-06-23

**Authors:** Marcin W. Wojewodzic

**Affiliations:** ^1^ Cancer Registry of Norway (Kreftregisteret), Institute of Population-Based Cancer Research, Etiology Group, NO-0304, Oslo, Norway.; ^2^ School of Biosciences, University of Birmingham, Birmingham, B15 2TT, United Kingdom.

**Keywords:** bacteriophages, bacterial infections, covid-19, phage display therapies

## Abstract

The pandemic of the coronavirus disease (Covid-19) has caused the death of at least 270,000 people as of the 8th of May 2020. This work stresses the potential role of bacteriophages to decrease the mortality rate of patients infected by the severe acute respiratory syndrome coronavirus 2 (SARS-CoV-2) virus. The indirect cause of mortality in Covid-19 is miscommunication between the innate and adaptive immune systems, resulting in a failure to produce effective antibodies against the virus on time. Although further research is urgently needed, secondary bacterial infections in the respiratory system could potentially contribute to the high mortality rate observed among the elderly due to Covid-19. If bacterial growth, together with delayed production of antibodies, is a significant contributing factor to Covid-19's mortality rate, then the additional time needed for the human body's adaptive immune system to produce specific antibodies could be gained by reducing the bacterial growth rate in the respiratory system of a patient. Independently of that, the administration of synthetic antibodies against SARS-CoV-2 viruses could potentially decrease the viral load. The decrease of bacterial growth and the covalent binding of synthetic antibodies to viruses should further diminish the production of inflammatory fluids in the lungs of patients (the indirect cause of death). Although the first goal could potentially be achieved by antibiotics, I argue that other methods may be more effective or could be used together with antibiotics to decrease the growth rate of bacteria, and that respective clinical trials should be launched.

Both goals can be achieved by bacteriophages. The bacterial growth rate could potentially be reduced by the aerosol application of natural bacteriophages that prey on the main species of bacteria known to cause respiratory failure and should be harmless to a patient. Independently of that, synthetically changed bacteriophages could be used to quickly manufacture specific antibodies against SARS-CoV-2. This can be done via a Nobel Prize awarded technique called “phage display.” If it works, the patient is given extra time to produce their own specific antibodies against the SARS-CoV-2 virus and stop the damage caused by an excessive immunological reaction.

## The Virus That Caused the Pandemic

The coronavirus pandemic has caused the death of more than 270,000 people, as reported by 8th May 2020 by the World Health Organization (WHO). The crisis we observe is the joint effect of globalization and the properties of the new virus (SARS-CoV-2), which causes the disease, Covid-19. SARS-CoV-2 stands for “Severe Acute Respiratory Syndrome COronaVirus 2” describing one of the most dangerous symptoms in Covid-19. Although there have been past warnings of the threat that respiratory targeting viruses pose,^1^ the SARS-CoV-2 virus has spread at an unprecedented rate and it is devastating our health and economy globally. We urgently need multiple approaches to tackle this crisis.

This short communication attempts to highlight the potential for the use of natural bacteriophages to decrease the mortality rate among patients infected by the SARS-CoV-2 virus. Covid-19 patients can develop SARS, leading to atypical pneumonia^2^ that is mediated by cytokine storms.^3^

## Possible Significance of Bacteria in Symptoms for Covid-19

The most probable entrance road of the SARS-CoV-2 to humans is the respiratory system, where the virus can disrupt its equilibrium.

The indirect cause of death in Covid-19 patients could be miscommunication between the innate and adaptive immunological systems.^4^ The adaptive immune response takes much longer than the innate immune response to begin effectively attacking a new pathogen. This means there is a period when only the innate immune system is fighting the infection and, in this period, the innate immune system's response can become too aggressive when faced with a high virus load, causing it to damage other systems. The growth of the virus causes the innate immune system to secrete inflammatory material (fluid and inflammatory cells) into the lungs. As a result, the lungs become filled with fluid reducing the body's ability to exchange gases.^4^

The debris of dying and virally infected human respiratory cells can become a substrate for bacteria growth, a side effect of the virus infection. This growth of bacteria then causes the innate immune system to secrete additional inflammatory material in nearby alveoli. Bacterial infections seem to provoke a further reaction of the innate immune system, and they may interact with virus infections.^5^ This process accelerates as the virus continues to attack lung cells, and it thus creates more cell debris substrate for the bacteria to feed on. This can result in the innate immune system adding too much inflammatory fluid to the lungs, inhibiting gas exchange and resulting in an urgent need for ventilation, and it can cause sepsis and death.

The delay (or failure) of the production of antibodies specific to the virus could explain why SARS-CoV-2 is so dangerous for the elderly. A recent detailed review on immunity in Covid-19 summarizes state-of-the-art knowledge of the host's immunological response to the virus, and it points out clear differences in disease progression between younger and older patients.^4^

**FIG. 1.**
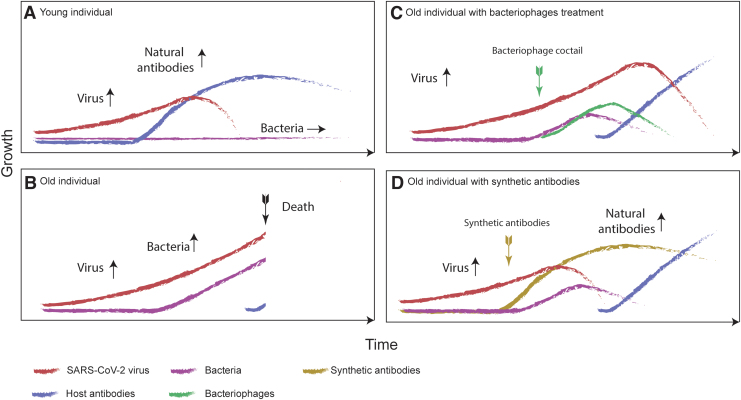
Theoretical time courses of the SARS-CoV-2 virus growth (red curves), bacterial growth (purple curves), and host antibody production (blue curves) for four scenarios. **(A)** A young healthy individual who has no problems developing antibodies to the virus infection. **(B)** An old individual who experiences delayed antibody production, resulting in bacterial growth as well as increased virus growth. **(C)** An old individual for whom a bacteriophage cocktail against bacterial growth was introduced as a part of standard therapy. Increase of bacteriophages is marked (green curve) with the time of treatment (green arrow). The relationship between bacteriophages and bacteria can be described by the Lotkka-Voltera population model. The viral load does not decrease until the body's natural antiviral antibodies are produced but more time is bought for this to happen. **(D)** An old individual for whom synthetic antibodies were introduced (brown curve), creating an immediate reduction in the viral load and once again buying time for the natural antibodies to be produced. SARS-CoV-2, severe acute respiratory syndrome coronavirus 2.

Immunosenescence (impairment of immune functions) can delay the production of antibodies and is usually expected in elderly patients (Fig. 1B),^6,7^ which might be a part of the cause for the high age-dependent mortality observed in Covid-19 patients (Fig. 1A, B). Although data for Covid-19 are still scarce, there is evidence that having previously contracted influenza predisposes the host to acquiring pneumococcal colonization^8,9^ and therefore there is a known mechanism for viral infections to cause bacterial colonization in the human respiratory system. Further, the co-occurrence of viruses and bacteria is well documented for other viruses.^10^

Although ecologists call this process a “succession,” medical doctors use the term “secondary infections.” For instance *Staphylococcus aureus*, *Staphylococcus pneumoniae* (pneumococcus), *Aerococcus viridans, Haemophilius influenza,* and *Moraxella catarrhalis* are bacteria typically found in influenza patients, as well as other respiratory commensals, which occasionally turn into pathogens causing infection.^11^

A recent review suggests that bacterial infections, including *Acinetobacter baumanii* and *Klebsiella pneumoniae*, have been documented in Covid-19 patients, especially in the intensive care unit setting.^2^ Non-survivors were more likely to have sepsis and secondary infection, although detailed bacteriology results were not reported. Secondary infections were also positively correlated with steroid administration.^2^

At least part of the high mortality rate attributed to Covid-19 could be due to bacterial infection of the respiratory system,^12,13^ although we still do not have an accurate estimate for the numbers. There might also be problems in producing reliable estimates for these numbers due to the overwhelming number of patients seen in clinics and the criteria for which patients are admitted to bacteriology tests, and at what point in the process. A recent report from Wuhan shows that at least 50% of patients dying developed secondary infections.^12^ The median time given for these secondary infections to develop is 17 days, although the range in time is quite large. It is plausible that bacterial infections begin to colonize before acute respiratory distress syndrome is developed.

In viral scenarios such as influenza, bacteria such as *Pseudomonas aeruginosa* are known to spread rapidly.^14,15^ In addition, the rapid and enormous response of the first-line, innate immunity system causes general inflammation that can change pulmonary structures (causing fibrosis), further reducing oxygen uptake and causing permanent damage to the respiratory tissue. This reaction can lead to the innate immunity system itself being the actual cause of death; however, the extent to which this reaction is caused by the body's response to the SARS-CoV-2 virus or to which it is caused by its response to infection by bacteria (such as *P. aeruginosa*) is not yet known and I postulate may differ over the course of the infection.

The interplay between the time taken for the human body to develop antiviral antibodies and the role of bacteria in the death of older individuals is also not yet well known for Covid-19.

## Integrative Approach Proposal

If bacterial growth, together with the delayed production of antibodies, is a significant contributing factor to Covid-19's mortality rate, then the additional time needed for the human body's adaptive immunity system to produce antibodies could be gained by reducing the bacterial growth rate in the respiratory system of the patient. If the growth of bacteria in lungs can be stopped, then the rate of liquid increase within the lungs should also decrease. However, as the growth of the virus is exponential, it might be necessary to decrease the viral load at the same time as the bacterial load to slow down the immunological response.

## Natural Bacteriophages' Potential—A Direct Weapon Against Bacteria

Bacteriophages are viruses that selectively attack specific species of bacteria and are otherwise harmless to animal cells, including humans. They were discovered 100 years ago by Frederick W. Twort and Félix d'Hérelle^16^ and are distributed throughout Earth's ecosystems^17^ and over a broad bacterial host range, including bacteria naturally found in humans.^18^

It has been shown that the attack of bacteriophages is specific, meaning that one species of bacteriophage targets only a single species of bacteria (or even a specific strain of one species).^19^ This specificity also points toward the “Red Queen” co-evolutionary process between these two players.^20,21^ The scenario of the attack is as follows: (1) The bacteriophage attaches itself to a susceptible bacterium, exclusively infects the host bacterial cell and (2) hijacks the bacterium's biochemical machinery to produce multiple copies of itself. (3) The bacterium then undergoes destruction (lysis) and new copies of the bacteriophage are released and infect, exclusively, other bacteria of the same species in the neighboring areas.

Despite this known interplay between bacteriophages and bacteria, research into bacteriophages and their potential medical applications was largely abandoned for many years due to “The Antibiotics Revolution.” Antibiotics were adopted as the main way of treating bacterial infections due primarily to the fact that they are general purpose, as opposed to bacteriophages that specifically target a single species of bacteria. Other advantages include the fact that antibiotics are usually fast acting, efficient, and relatively cheap to manufacture. However, there are several drawbacks as well to the use of antibiotics. One of these is that, unlike bacteriophages, antibiotics can destroy beneficial bacteria in addition to harmful ones.^22^ More importantly, the overuse of antibiotics can cause bacteria to evolve resistances to them, resulting in antibiotic-immune “superbugs.”^23,24^

In the current Covid-19 pandemic, around 70% of hospitalized COVID-19 patients worldwide receive antibiotics as part of their treatment.^25^ This raises the danger of the emergence of antibiotic-resistant strains of bacteria even higher and creates an even greater need for the development of alternative strategies to fight bacterial infections. Unlike antibiotics, bacteriophage treatments would be far less susceptible to the development of resistances, as the bacteriophage itself can also adapt to overcome any resistance that the bacteria develop.^26^

It has also been suggested that the presence of bacteriophages can have positive effects on human health and patient recovery, suggesting that bacteriophages are to some extent responsible for homeostasis of the microbiota.^27^ For instance, a group investigating alternative treatments for *Clostridium difficile*, a bacteria that can infect the bowel and cause diarrhea, has identified a large set of bacteriophages that are effective at killing this pathogen.^28^ This method is now being transformed into a therapeutic treatment. We can find more examples of how bacteriophages are being used for human or animal models, in addition to different bioengineering methods using bacteriophages that are currently being developed.^29–31^

## Bacteriophages Used for Accelerated Therapeutic Antibody Production Against the Virus

Despite the fact that bacteriophages' potential to fight bacterial infections has only recently been rediscovered, they were successfully used as tools at the molecular level, leading to Nobel Prize awards.^32^

Using a technique called phage display, bacteriophages have the potential to quickly produce recombinant antibodies.^33^ This technique of producing antibodies was developed for MERS-CoV and successfully applied.^34^ In phage display, techniques blocking ACE2 interaction could be engineered from the serum of immune patients. The Yin-Yang biopanning method highlights the possibility of utilizing crude antigens for the isolation of monoclonal antibodies by phage display. Before this, artificial antibody production was primarily done by using animals; however, this is both slower and less cost effective than using bacteriophage display techniques.^35^ Another benefit of this method is that monoclonal antibodies produced by bacteriophage display techniques can be humanized.^36^

The use of antibody therapy for the control of viral diseases has already been reviewed and some therapies have been approved for human testing.^37^ As an example, the company ProteoGenix launched accelerated therapeutic antibody discovery by screening a naive antibody human library (LiAb-SFMAX™, scFv, Fab, IgG) or an immune human antibody library (obtained from the plasma of COVID-19 survivors) by using the phage display technique (https://bit.ly/2LlOsVQ). This demonstrates that accelerated therapeutic antibody discovery is highly feasible.

Therefore, there are two main ways that bacteriophages could be used to decrease the mortality rate of the Covid-19 pandemic. They can be used to decrease the population of bacteria in a patient's respiratory system and/or bacteriophage display techniques can be used to efficiently manufacture synthetic antibodies against SARS-CoV-2 (Fig. 1D).

I propose a series of clinical trials for the use of cocktails of bacteriophages (that target the main species of bacteria known to cause respiratory problems) in treating Covid-19 patients and/or the use of phage display techniques to create synthetic antibodies that target SARS-CoV-2 in the early stages of infection.

## Further Considerations for Bacteriophage Therapy—Bacteriophages as Killers

The bacterial growth rate could potentially be reduced by the aerosol application of bacteriophages that prey on the main species of bacteria known to cause respiratory failures (Fig. 1C). This can occur in a self-regulatory manner, similar to ecological prey–predator regulation. The exponential growth of the bacteriophage population (limited primarily by the population of the bacteria it preys on) should allow for a fast clearance, especially in cases where the bacterial population has already grown significantly. The relationship can be described by Lotka-Volterra or Kill-the-Winner population model.^38–40^

In fact, we can already find evidence in literature that pneumonia could be cured by nebulized bacteriophages.^41^ Prophylactically administered bacteriophages reduced lung bacterial burdens and improved survival of antibiotic-resistant *S. aureus* infected animals in the context of ventilator-associated pneumonia. If needed, a selection of bacteriophages and optimal target bacteria could be quickly identified by a group of experts as the species of bacteria that commonly cause respiratory problems are well known and a bacteriophage that preys on a specific species can be quickly identified by screening methods.^42^ If needed, quantitative microbiome sequencing could potentially be used.^43^

There are assumptions that need to be met during the clinical trials for the approach to work. (1) The cohort has to be chosen to have a high probability of developing bacterial infections. (2) It should be ensured to have the correct choice of bacteriophages that both target the optimal bacteria candidates and are most effective at reducing that bacteria's population growth. (3) The bacteriophages should not interfere with the patient's innate or adaptive immune system. (4) The patient does not have antibodies toward bacteriophages used, nor develops any antibodies toward bacteriophages to clear off the bacteriophage earlier than to SARS-CoV-2. We know from bacteriophage therapy in the pneumonia system that the rapid lysis of bacteria by bacteriophages *in vivo* does not increase the innate inflammatory response compared with antibiotic treatment.^44^ This is a promising finding and there seemed to be positive effects on the patient's immune system.^45^ (5) Another obstacle could be a risk of a species of bacteria developing resistance to the bacteriophage, according to the co-evolutionary process mentioned. However, this would be much less serious than the antibiotic resistance problem as it would only reduce the effectiveness of that one bacteriophage and there is the possibility of the bacteriophage also adapting to overcome any resistance to it. (6) Finally, bacteriophages are so specific to one species of bacteria, and there is very little chance of the bacteriophage damaging any beneficial bacteria, but this should still be verified in clinical trials. It has to be noted that the point here is to decrease bacterial growth in critical time and therefore allow the patient more time to recover from the Covid-19 infection.

## Decreasing the Population Growth Rate of Bacteria

The response to antibiotics may be slower or smaller than expected. This may be due to both antibiotic-resistant strains and slow diffusion rate of the antibiotics in that area due to bacterial biofilm formation.^46^ Also, in some cases, the penetration of antibiotics into target tissues is also dependent on the tissue type that was shown for lungs in tuberculosis scenarios.^47^ It has been shown that the sites of mycobacterial infection in the lungs of patients have complex structures and poor vascularization, which obstructs drug distribution to these hard-to-reach and hard-to-treat disease sites, further leading to suboptimal drug concentrations. Because of this, there is the potential for the use of bacteriophages (entering patients' respiratory systems in a different way and acting differently to antibiotics) to decrease the mortality rate of patients infected by the SARS-CoV-2 virus.

Intensive use of antibiotics targeting Covid-19 in clinics can further lead to bacterial resistance spreading in the hospitals. Using bacteriophages could take pressure off this problem. This could also shed light on the use of bacteriophages to decrease this problem in post–Covid-19 scenarios.

## Decrease the Viral Load by Using Synthetic Antiviral Antibodies

There are also assumptions that need to be met during the clinical trials for the second approach to work. (1) The cohort has to be chosen to have a bad prognosis (age >80) and high viral load; (2) ensuring the correct choice of antibody that targets the virus epitope and nothing else in the human body; (3) the antibody should not cause failure of the immune system (anaphylactic shock); (4) the dose and frequency should be mathematically modeled; and (5) the delivery system should be efficient.

## Gaps in Knowledge

Before choosing the candidate bacteriophages, careful literature studies will need to be done to check for potential known interactions. For example, it has been shown that some bacteria can produce a biofilm when exposed to their relevant bacteriophages,^48^ which could be an obstacle for the development of these methods as a treatment for Covid-19 patients. Although most bacteriophages kill their bacterial hosts, others can live inside the microbes without killing them.^49^ Also, lessons from recent studies need to be carefully followed. For instance, complex immune dysregulation in Covid-19 patients with severe respiratory failure has been observed.^50^

During the writing of this communication, the first immunological reviews were published, in which the authors identified major gaps in knowledge that need to be addressed by the scientific community.^4^ It is unknown how this may complicate any treatment and further investigation is needed.

## High Gain Approach

However, if a treatment using bacteriophages therapy can be developed it is likely to prove practical as they can be produced both quickly and cheaply. Production of antibodies from the phage display techniques will have some costs of production but, owing to recent progress, the development should be simple. Bacteriophages can also be stored and transported easily. I believe that bacteriophages have the potential to be a practical tool in mitigating the SARS-CoV-2 pandemic, especially in patients with secondary bacterial infection and high viral load. I believe that it is unlikely to have any significant side effects, and that it has the potential to save a great number of lives. The beauty of nature is that although it can kill us, it can also come to our rescue.

## References

[1] Sung JJY. Will the SARS epidemic recur? Sev Acute Respir Syndr. 2004;251–254.

[2] Cevik M, Bamford C, Ho A. COVID-19 pandemic—A focused review for clinicians. Clin Microbiol Infect. 2020. DOI: 10.1016/j.cmi.2020.04.023. [Epub ahead of print]. PMC7182753 32344166

[3] Huang C, Wang Y, Li X, et al. Clinical features of patients infected with 2019 novel coronavirus in Wuhan, China. Lancet. 2020;395:497–506. 31986264 10.1016/S0140-6736(20)30183-5 PMC7159299

[4] Tay MZ, Poh CM, Rénia L, et al. The trinity of COVID-19: Immunity, inflammation and intervention. Nat Rev Immunol. April 28, 2020. DOI: 10.1038/s41577-020-0311-8. [Epub ahead of print]. PMC7187672 32346093

[5] Shi Z, Gewirtz A. Together forever: Bacterial–viral interactions in infection and immunity. Viruses. 2018;10:122. 10.3390/v10030122 PMC5869515 29534424

[6] Stiasny K, Aberle JH, Keller M, et al. Age affects quantity but not quality of antibody responses after vaccination with an inactivated flavivirus vaccine against tick-borne encephalitis. PLoS One. 2012;7:e34145. 22461903 10.1371/journal.pone.0034145 PMC3312914

[7] Ventura MT, Casciaro M, Gangemi S, et al. Immunosenescence in aging: Between immune cells depletion and cytokines up-regulation. Clin Mol Allergy. 2017;15:21. 29259496 10.1186/s12948-017-0077-0 PMC5731094

[8] Grijalva CG, Griffin MR, Edwards KM, et al. The role of influenza and parainfluenza infections in nasopharyngeal pneumococcal acquisition among young children. Clin Infect Dis. 2014;58:1369–1376. 24621951 10.1093/cid/ciu148 PMC4001292

[9] Butler JC, Schuchat A. Epidemiology of pneumococcal infections in the elderly. Drugs Aging. 1999;15:11–19. 10690791 10.2165/00002512-199915001-00002

[10] Almand EA, Moore MD, Jaykus L-A. Virus-bacteria interactions: An emerging topic in human infection. Viruses. March 21, 2017;9. DOI: 10.3390/v9030058. [Epub ahead of print]. PMC5371813 28335562

[11] Bosch AATM, Biesbroek G, Trzcinski K, et al. Viral and bacterial interactions in the upper respiratory tract. PLoS Pathog. 2013;9:e1003057. 23326226 10.1371/journal.ppat.1003057 PMC3542149

[12] Zhou F, Yu T, Du R, et al. Clinical course and risk factors for mortality of adult inpatients with COVID-19 in Wuhan, China: A retrospective cohort study. Lancet. 2020;395:1054–1062. 32171076 10.1016/S0140-6736(20)30566-3 PMC7270627

[13] Dagur HS. Genome organization of Covid-19 and emerging severe acute respiratory syndrome Covid-19 outbreak: A pandemic. Eurasian J Med Oncol. 2020. DOI: 10.14744/ejmo.2020.96781. [Epub ahead of print].

[14] Langan KM, Kotsimbos T, Peleg AY. Managing *Pseudomonas aeruginosa* respiratory infections in cystic fibrosis. Curr Opin Infect Dis. 2015;28:547–556. 26524327 10.1097/QCO.0000000000000217

[15] Seki M, Higashiyama Y, Tomono K, et al. Acute infection with influenza virus enhances susceptibility to fatal pneumonia following *Streptococcus pneumoniae* infection in mice with chronic pulmonary colonization with *Pseudomonas aeruginosa*. Clin Exp Immunol. 2004;137:35–40. 15196241 10.1111/j.1365-2249.2004.02481.x PMC1809089

[16] Radetsky P. The Invisible Invaders: Viruses and the Scientists who Pursue Them. Boston: Back Bay Books; 1994.

[17] Al-Shayeb B, Sachdeva R, Chen L-X, et al. Clades of huge phages from across Earth's ecosystems. Nature. 2020;578:425–431. 32051592 10.1038/s41586-020-2007-4 PMC7162821

[18] Shkoporov AN, Hill C. Bacteriophages of the human gut: The “Known Unknown” of the microbiome. Cell Host Microbe. 2019;25:195–209. 30763534 10.1016/j.chom.2019.01.017

[19] Koskella B, Meaden S. Understanding bacteriophage specificity in natural microbial communities. Viruses. 2013;5:806–823. 23478639 10.3390/v5030806 PMC3705297

[20] Mueller L. 1973 The red queen hypothesis. In: Dulberger A, Avise J; eds. Conceptual Breakthroughs in Evolutionary Ecology. Cambridge, MA: Elsevier, Academic Press; 2020: 85–86.

[21] Papkou A, Guzella T, Yang W, et al. The genomic basis of Red Queen dynamics during rapid reciprocal host–pathogen coevolution. Proc Natl Acad Sci. 2019;116:923–928. 30598446 10.1073/pnas.1810402116 PMC6338873

[22] Willing B, Russell S, Finlay B. Shifting the balance: Antibiotic effects on host–microbiota mutualism. Nat Rev Microbiol. 2011;9:233–243. 21358670 10.1038/nrmicro2536

[23] Salyers A. The problem of antibiotic resistance. Ann Rev Microbiol. 2003. DOI: 10.1146/annurev.micro.58.030603. [Epub ahead of print].

[24] Aslam B, Wang W, Arshad MI, et al. Antibiotic resistance: A rundown of a global crisis. Infect Drug Resist. 2018;11:1645–1658. 30349322 10.2147/IDR.S173867 PMC6188119

[25] Hsu J. How covid-19 is accelerating the threat of antimicrobial resistance. BMJ. 2020;369. DOI: 10.1136/bmj.m1983. 32423901

[26] Czaplewski L, Bax R, Clokie M, et al. Alternatives to antibiotics-a pipeline portfolio review. Lancet Infect Dis. 2016;16:239–251. 26795692 10.1016/S1473-3099(15)00466-1

[27] Blanco-Picazo P, Fernández-Orth D, Brown-Jaque M, et al. Unravelling the consequences of the bacteriophages in human samples. Sci Rep. 2020;10:6737. 32317653 10.1038/s41598-020-63432-7 PMC7174282

[28] Nale JY, Redgwell TA, Millard A, et al. Efficacy of an optimised bacteriophage cocktail to clear *Clostridium difficile* in a batch fermentation model. Antibiotics (Basel). February 13, 2018;7. DOI: 10.3390/antibiotics7010013. [Epub ahead of print]. PMC5872124 29438355

[29] Nir-Paz R, Gelman D, Khouri A, et al. Successful treatment of antibiotic-resistant, poly-microbial bone infection with bacteriophages and antibiotics combination. Clin Infect Dis. 2019;69:2015–2018. 30869755 10.1093/cid/ciz222

[30] Cafora M, Deflorian G, Forti F, et al. Phage therapy against *Pseudomonas aeruginosa* infections in a cystic fibrosis zebrafish model. Sci Rep. 2019;9. DOI: 10.1038/s41598-018-37636-x. [Epub ahead of print]. PMC6365511 30728389

[31] Peng H, Borg RE, Dow LP, et al. Controlled phage therapy by photothermal ablation of specific bacterial species using gold nanorods targeted by chimeric phages. Proc Natl Acad Sci U S A. 2020;117:1951–1961. 31932441 10.1073/pnas.1913234117 PMC6994977

[32] Barderas R, Benito-Peña E. The 2018 Nobel Prize in Chemistry: Phage display of peptides and antibodies. Anal Bioanal Chem. 2019;411:2475–2479. 30888467 10.1007/s00216-019-01714-4

[33] Shukra AM, Sridevi NV, Dev Chandran, et al. Production of recombinant antibodies using bacteriophages. Eur J Microbiol Immunol. 2014;4:91–98. 10.1556/EuJMI.4.2014.2.1 PMC4029287 24883194

[34] Lim CC, Woo PCY, Lim TS. Development of a phage display panning strategy utilizing crude antigens: Isolation of MERS-CoV nucleoprotein human antibodies. Sci Rep. 2019;9:6088. 30988390 10.1038/s41598-019-42628-6 PMC6465254

[35] Hentrich C, Ylera F, Frisch C, et al. Monoclonal antibody generation by phage display. In: Vashist SK, Luony JHT; eds. Handbook of Immunoassay Technologies. Cambridge, MA: Elsevier, Academic Press; 2018: 47–80.

[36] Frenzel A, Kügler J, Helmsing S, et al. Designing human antibodies by phage display. Transfus Med Hemother. 2017;44:312–318. 29070976 10.1159/000479633 PMC5649246

[37] Dibo M, Battocchio EC, Dos Santos Souza LM, et al. Antibody therapy for the control of viral diseases: An update. Curr Pharm Biotechnol. 2019;20:1108–1121. 31400263 10.2174/1389201020666190809112704

[38] Wangersky PJ. Lotka-Volterra population models. Ann Rev Ecol Syst. 1978;9:189–218.

[39] Maslov S, Sneppen K. Population cycles and species diversity in dynamic Kill-the-Winner model of microbial ecosystems. Sci Rep. 2017;7:39642. 28051127 10.1038/srep39642 PMC5209715

[40] Eriksen RS, Mitarai N, Sneppen K. Sustainability of spatially distributed bacteria-phage systems. Sci Rep. 2020;10:3154. 32081858 10.1038/s41598-020-59635-7 PMC7035299

[41] Prazak J, Valente L, Iten M, et al. Nebulized bacteriophages for prophylaxis of experimental ventilator-associated pneumonia due to methicillin-resistant Staphylococcus aureus. Crit Care Med. April 16, 2020. DOI: 10.1097/CCM.00000000000043521. [Epub ahead of print]. 32304419

[42] Rajnovic D, Muñoz-Berbel X, Mas J. Fast phage detection and quantification: An optical density-based approach. PLoS One. 2019;14:e0216292. 31071103 10.1371/journal.pone.0216292 PMC6508699

[43] Barlow JT, Bogatyrev SR, Ismagilov RF. A quantitative sequencing framework for absolute abundance measurements of mucosal and lumenal microbial communities. DOI: 10.1101/2020.02.28.970087. PMC7244552 32444602

[44] Dufour N, Delattre R, Chevallereau A, et al. Phage therapy of pneumonia is not associated with an overstimulation of the inflammatory response compared to antibiotic treatment in mice. Antimicrob Agents Chemother. August 2019;63. DOI: 10.1128/AAC.00379-19. [Epub ahead of print]. PMC6658787 31182526

[45] Górski A, Międzybrodzki R, Jończyk-Matysiak E, et al. Phage-specific diverse effects of bacterial viruses on the immune system. Future Microbiol. 2019;14:1171–1174. 31535921 10.2217/fmb-2019-0222 PMC6802706

[46] Sharma D, Misba L, Khan AU. Antibiotics versus biofilm: An emerging battleground in microbial communities. Antimicrob Resist Infect Control. 2019;8:76. 31131107 10.1186/s13756-019-0533-3 PMC6524306

[47] Strydom N, Gupta SV, Fox WS, et al. Tuberculosis drugs' distribution and emergence of resistance in patient's lung lesions: A mechanistic model and tool for regimen and dose optimization. PLoS Med. 2019;16:e1002773. 30939136 10.1371/journal.pmed.1002773 PMC6445413

[48] Secor PR, Sweere JM, Michaels LA, et al. Filamentous bacteriophage promote biofilm assembly and function. Cell Host Microbe. 2015;18:549–559. 26567508 10.1016/j.chom.2015.10.013 PMC4653043

[49] Reardon S. Virus tricks the immune system into ignoring bacterial infections. Nature. March 28, 2019. DOI: 10.1038/d41586-019-00991-4. [Epub ahead of print]. 32218544

[50] Giamarellos-Bourboulis EJ, Netea MG, Rovina N, et al. Complex immune dysregulation in COVID-19 patients with severe respiratory failure. Cell Host Microbe. April 17, 2020. DOI: 10.1016/j.chom.2020.04.009. [Epub ahead of print]. PMC7172841 32320677

